# Convergent evolution of gene expression in two high-toothed stickleback populations

**DOI:** 10.1371/journal.pgen.1007443

**Published:** 2018-06-13

**Authors:** James C. Hart, Nicholas A. Ellis, Michael B. Eisen, Craig T. Miller

**Affiliations:** 1 Department of Molecular and Cell Biology, University of California-Berkeley, CA, United States of America; 2 Howard Hughes Medical Institute, University of California, Berkeley, CA, United States of America; University of Illinois, UNITED STATES

## Abstract

Changes in developmental gene regulatory networks enable evolved changes in morphology. These changes can be in *cis* regulatory elements that act in an allele-specific manner, or changes to the overall *trans* regulatory environment that interacts with *cis* regulatory sequences. Here we address several questions about the evolution of gene expression accompanying a convergently evolved constructive morphological trait, increases in tooth number in two independently derived freshwater populations of threespine stickleback fish (*Gasterosteus aculeatus*). Are convergently evolved *cis* and/or *trans* changes in gene expression associated with convergently evolved morphological evolution? Do *cis* or *trans* regulatory changes contribute more to gene expression changes accompanying an evolved morphological gain trait? Transcriptome data from dental tissue of ancestral low-toothed and two independently derived high-toothed stickleback populations revealed significantly shared gene expression changes that have convergently evolved in the two high-toothed populations. Comparing *cis* and *trans* regulatory changes using phased gene expression data from F1 hybrids, we found that *trans* regulatory changes were predominant and more likely to be shared among both high-toothed populations. In contrast, while *cis* regulatory changes have evolved in both high-toothed populations, overall these changes were distinct and not shared among high-toothed populations. Together these data suggest that a convergently evolved trait can occur through genetically distinct regulatory changes that converge on similar *trans* regulatory environments.

## Author summary

Convergent evolution, where a similar trait evolves in different lineages, provides an opportunity to study the repeatability of evolution. Convergent morphological evolution has been well studied at multiple evolutionary time scales ranging from ancient, to recent, such as the gain in tooth number in freshwater stickleback fish. However, much less is known about the accompanying evolved changes in gene regulation during convergent evolution. Here we compared evolved changes in gene expression in dental tissue of ancestral low-toothed marine fish to fish from two independently derived high-toothed freshwater populations. We also partitioned gene expression changes into those affecting a gene’s regulatory elements (*cis*), and those affecting the overall regulatory environment (*trans*). Both freshwater populations have evolved similar gene expression changes, including a gain of expression of putative dental genes. These similar gene expression changes are due mainly to shared changes to the *trans* regulatory environment, while the *cis* changes are largely population specific. Thus, during convergent evolution, overall similar and perhaps predictable transcriptome changes can evolve despite largely different underlying genetic bases.

## Introduction

Development is controlled by a complex series of interlocking gene regulatory networks. Much of this regulation occurs at the level of transcription initiation, where *trans* acting factors bind to *cis* regulatory elements to control their target gene’s expression [1,2]. Evolved changes in an organism's morphology are the result of changes in this developmental regulatory landscape. It has been proposed that the genetic bases of many of these evolved changes are mutations within the *cis*-regulatory elements of genes [3–5]. Indeed, recent work in evolutionary genetics suggests the molecular bases of a diverse array of traits from *Drosophila* wing spots [6] to mouse pigmentation [7] to stickleback armored plate number [8,9] and size [10] are changes in the activity of *cis*-regulatory elements.

Evolved changes in gene expression can be divided into two broad regulatory classes. *Cis* regulatory changes can occur within the proximal promoter [11], distal enhancer [12], or the gene body itself [13], and result in allele-specific gene expression differences in hybrid diploids [14]. *Trans* regulatory changes modify the overall regulatory environment [15,16], but are usually genetically unlinked to the expression change, and do not result in allele-specific expression in hybrid diploids. For any gene with an evolved expression difference, the total evolved gene expression difference can be partitioned into changes in *cis* and *trans* by quantifying expression differences between two populations and also testing for expression differences between alleles in F1 hybrids between the two populations [14]. As both alleles in F1 hybrids animals are exposed to the same regulatory environment, any difference in their expression must be due to a *cis*-regulatory change. Several studies have attempted to characterize evolved *cis* and *trans*-regulatory changes at a transcriptome-wide level [17–21]. Though the relative contribution of *cis* and *trans* regulatory changes varies extensively among studies, *cis* changes have been found to dominate [17,18,21] or at least be approximately equivalent to *trans* changes [19,20,22]. Additionally, compensatory changes (*cis* and *trans* changes in opposing directions) have been found to be enriched over neutral models [17,18], showing evidence for selection for stable gene expression levels. However, none of these studies examined contribution of *cis* and *trans* gene expression changes during convergent morphological evolution.

Populations evolve new traits following a shift to a novel environment, due to a mixture of drift and selection. Truly adaptive traits can often be repeatedly observed in multiple populations following a similar ecological shift. Threespine sticklebacks are an excellent system for the study of evolved changes in phenotypes, including gene expression [23–27]. Marine sticklebacks have repeatedly colonized freshwater lakes and streams along the coasts of the Northern hemisphere [28]. Each of these freshwater populations has independently adapted to its new environment; however, several morphological changes, including a loss in armored plates and a gain in tooth number, are shared among multiple newly derived populations [29,30]. The repeated evolution of lateral plate loss is due to repeated selection of a standing variant regulatory allele of the *Eda* gene within marine populations [8,9] and genome sequencing studies found over a hundred other shared standing variant alleles present in geographically diverse freshwater populations [31]. These studies suggest the genetic basis of freshwater adaptation might typically involve repeated reuse of the same standing variants to evolve the same adaptive freshwater phenotype.

However, more recent evidence has shown that similar traits have also evolved through different genetic means in freshwater stickleback populations. A recent study which mapped the genetic basis of a gain in pharyngeal tooth number in two independently derived freshwater populations showed a largely non-overlapping genetic architecture [30]. Another study using three different independently derived benthic (adapted to the bottom of a lake) populations showed that, even when adapting to geographically and ecologically similar environments, the genetic architecture of evolved traits is a mix of shared and unique changes [32]. Even in cases where the same gene is targeted by evolution in multiple populations (the loss of *Pitx1* expression resulting in a reduction in pelvic spines), the individual mutations are often independently derived [33,34]. All of these genomic scale studies have looked at the genetic control of morphological changes, while the extent and nature of genome-wide gene expression changes has been less studied. It remains an open question as to whether similar gene expression patterns evolve during the convergent evolution of morphology, and if so, to what extent those potential shared gene expression changes are due to shared *cis* or *trans* changes.

Teeth belong to a class of vertebrate epithelial appendages (including mammalian hair) that develop from placodes, and have long served as a model system for studying organogenesis and epithelial-mesenchymal interactions in vertebrates [35]. Odontogenesis is initiated and controlled by complex interactions between epithelial and mesenchymal cell layers, and involves several deeply conserved signaling pathways [36–38]. Sticklebacks retain the ancestral jawed vertebrate condition of polyphyodonty, or continuous tooth replacement, and offer an emergent model system for studying tooth replacement. Previous work has supported the hypothesis that two independently derived freshwater stickleback populations have evolved an increase in tooth replacement rate, potentially mediated through differential odontogenic stem cell dynamics [30]. Recent studies have found teeth and taste bud development to be linked, with one study supporting a model where teeth and taste buds are copatterned from a shared oral epithelial source [39], and another study supporting a model where teeth and taste buds share a common progenitor stem cell pool [40].

We sought to examine the evolution of the regulatory landscape controlling stickleback tooth development and replacement. Using high-throughput RNA sequencing (RNA-seq) in parental non-hybrid fish, we found that two independently derived high-toothed freshwater populations display highly convergent gene expression changes, especially in orthologs of known tooth-expressed genes in other vertebrates, likely reflecting the convergently evolved tooth gain phenotype and the deep homology of teeth across all jawed vertebrates. We also quantitatively partitioned these evolved gene expression changes into *cis* and *trans* regulatory changes [14,19] in both populations at a transcriptome-wide level using RNA-seq on F1 marine-freshwater hybrids. We found that *trans* regulatory changes predominate evolved changes in gene expression in dental tissue. Additionally, we found that the *trans* regulatory changes are more likely to be shared between the freshwater populations than the *cis* regulatory changes. Thus, similar downstream transcription networks controlling tooth development and replacement have convergently evolved largely through different upstream genetic regulatory changes.

## Results

### Convergent evolution of tooth gain in two freshwater populations

**Fig 1:**
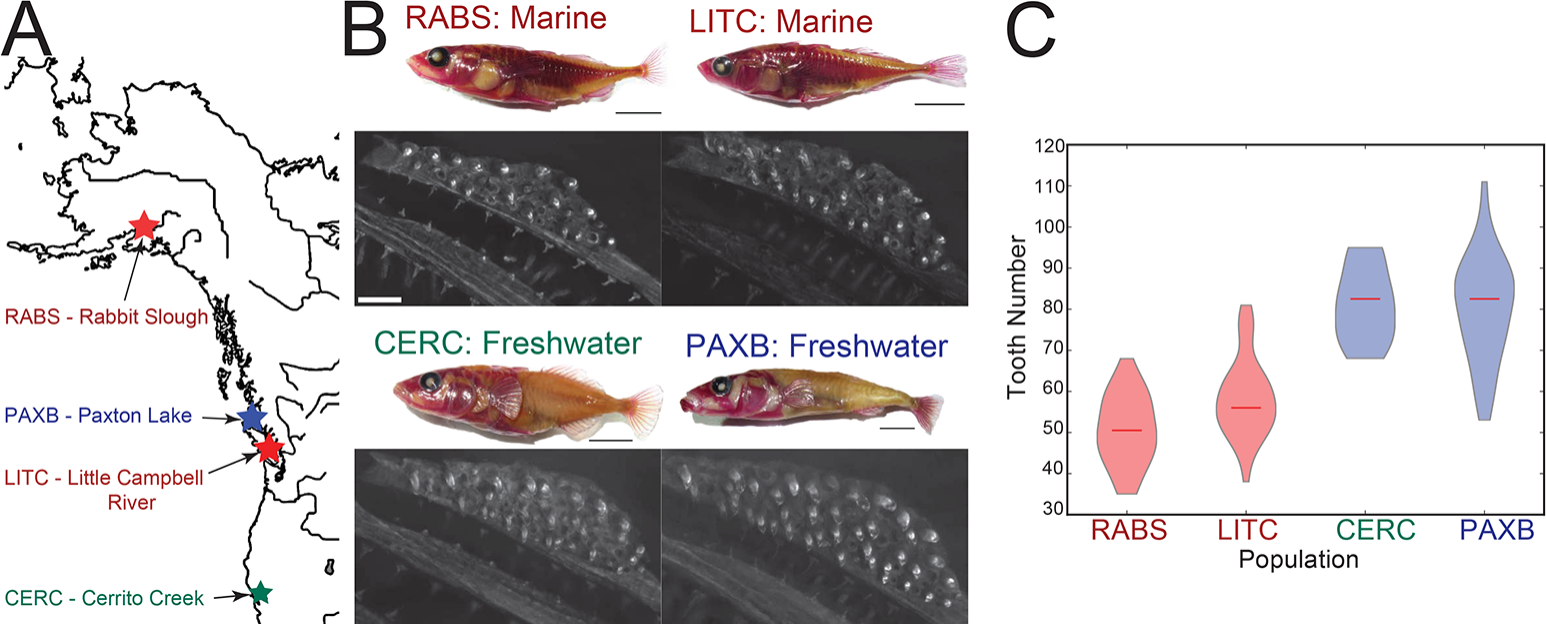
Evolved tooth gain in two freshwater populations. (A) Stickleback population locations. (B) Representative Alizarin red stained adult lab-reared sticklebacks (top, scale bars = 1 cm) and dissected ventral pharyngeal tooth plates (scale bars = 100μm). (C) Total ventral pharyngeal tooth number of 35–50 millimeter standard length lab-reared adult fish from each population. N = 44,52,12,32 for RABS_M_, LITC_M_, CERC_FW_, and PAXB_FW_, respectively.

To test whether multiple freshwater populations have evolved increases in tooth number compared to multiple ancestral marine populations [30,41], we quantified total ventral pharyngeal tooth number of lab reared sticklebacks from four distinct populations: (1) a marine population from the Little Campbell river (LITC_M_) in British Columbia, Canada, (2) a second marine population from Rabbit Slough (RABS_M_) in Alaska, USA, (3) a benthic freshwater population from Paxton Lake (PAXB_FW_) in British Columbia, Canada, and (4) a second freshwater population from Cerrito Creek (CERC_FW_) in California, USA (Fig 1A and 1B). Freshwater fish from both populations had more pharyngeal teeth than marine fish at this 35-50mm standard length (SL) stage, consistent with previous findings [30,41] of increases in tooth number in freshwater sticklebacks (Fig 1B and 1C, S1 Table).

To estimate the genomic relatedness of these populations, we resequenced the genomes of three marine and six freshwater sticklebacks from the four different populations (S2 Table). We aligned the resulting reads (mean of ~53 million reads per sample, see Methods and S2 Table) to the stickleback reference genome [31] using Bowtie2 [42], and called 8.3 million (see Methods) variants using the Genome Analysis Toolkit (GATK) [43–45]. As it has been previously shown that Pacific marine stickleback populations are an outgroup to freshwater populations from Canada (PAXB_FW_) and California (CERC_FW_) [31], we hypothesized the two high-toothed populations would be more related to each other genomically than either marine population. A phylogeny constructed using a down-sampled set of 67.5 thousand genome-wide variants (see Methods) cleanly separated freshwater populations from each other and from marine fish (S1A Fig). Principal component analysis using 1.7 million filtered genome-wide variants (see Methods) revealed that the first principle component explains nearly half (41.4%) of the overall variance and separates PAXB_FW_ sticklebacks from both CERC_FW_ and marine fish (S1B Fig), representing the independent evolution of PAXB_FW_ genomes. The second principal component separated both freshwater populations from marine populations, showing partially shared freshwater genome evolution. These results further support the model that populations of freshwater sticklebacks used a combination of shared and independent genetic changes [31,32] when evolving a set of similar morphological changes in response to a new environment.

### Convergent evolution of gene expression

**Fig 2:**
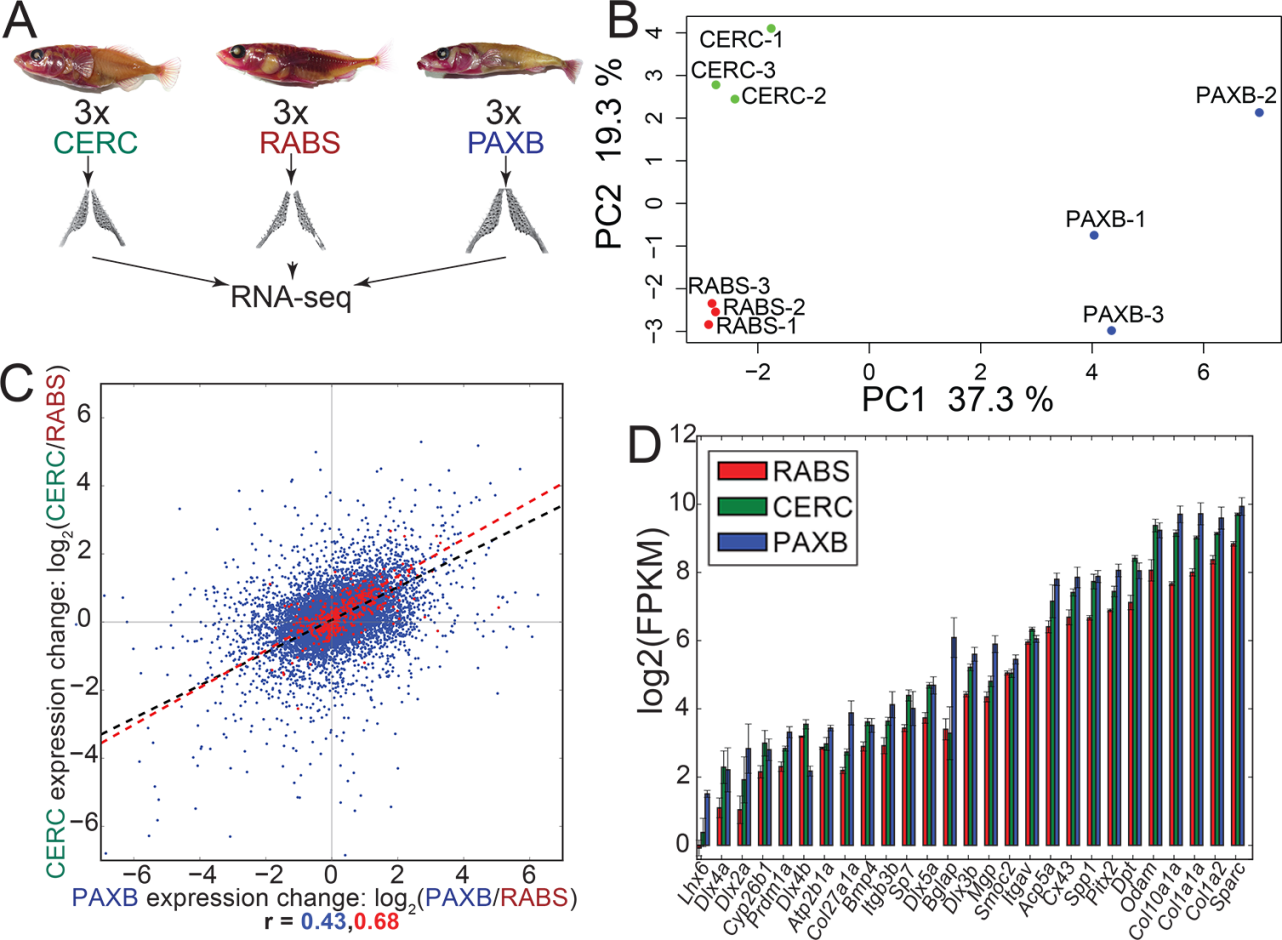
Convergent evolution of gene expression in dental tissue. (A) Ventral pharyngeal tooth plates from three different populations were dissected and gene expression quantified by RNA-seq. (B) Principal component analysis of dental tissue gene expression shows population specific expression profiles. (C) Freshwater dental tissue exhibited correlated gene expression changes for all genes (blue), with increased correlation observed for orthologs of genes known to be expressed during mammalian tooth development (red). (D) Expression of genes annotated as expressed in zebrafish teeth (zfin.org) which were significantly upregulated in one or both freshwater populations.

As morphological changes are often the result of changes in gene expression patterns and levels, we sought to identify evolved changes in gene expression during tooth development at stages soon after the evolved differences emerge [41]. We quantified gene expression in ventral pharyngeal dental tissue for three females each from the two high-toothed freshwater (PAXB_FW_ and CERC_FW_) and Alaskan (RABS_M_) low-toothed marine populations using RNA-seq (Fig 2A, S3 and S4 Tables). Principal component (PC) analysis of the resulting gene expression matrix showed a clustering of gene expression by population, with the first PC separating PAXB_FW_ samples, and the second PC separating both PAXB_FW_ and CERC_FW_ samples from marine, similar to the PC analysis of the genome-wide variants (Fig 2B) [46].

Given the convergently evolved morphological change of increases in tooth number, we hypothesized that convergent evolution has occurred at the gene expression level in freshwater dental tissue. To test this hypothesis, we performed a differential expression analysis, defining evolved changes in gene expression as changes found to be significant in a differential expression analysis using cuffdiff2 [47]. We compared evolved change in gene expression in PAXB_FW_ dental tissue (PAXB_FW_ expression vs marine) to the evolved change in CERC_FW_ dental tissue (CERC_FW_ expression vs marine). We found 6,693 and 3,501 genes (out of a total of 22,442) with significant (as determined by cuffdiff2 [47], see Methods) evolved expression changes in PAXB_FW_ and CERC_FW_ respectively. Of these genes with evolved expression changes, 2,223 were called differentially expressed in both populations, with 1,898 (85%) showing expression changes in the same direction relative to marine.

At a genome-wide level, correlated changes in gene expression levels have evolved in the two high-toothed freshwater populations (Fig 2C, Spearman's r = 0.43). We next asked if orthologs of genes implicated in tooth development in other vertebrates showed an increase in correlated evolved expression changes. We compared the gene expression changes of stickleback orthologs of genes in the BiteIt (http://bite-it.helsinki.fi/) [48] or ToothCODE (http://compbio.med.harvard.edu/ToothCODE/) [36] databases (hereafter referred to as the “BiteCode” gene set, S5 Table), two databases of genes implicated in mammalian tooth development. Consistent with the conserved roles of gene regulatory networks regulating mammalian and fish teeth [49–52] and the major evolved increases in tooth number in both freshwater populations (Fig 1C), these predicted dental genes showed an increase in their correlated evolved gene expression change (Fig 2C red points, Spearman's r = 0.68), and tended to have an overall increase in gene expression (S2 Fig, *P* = 7.36e-6, GSEA, see methods). This correlation coefficient was higher than any observed in over 100,000 bootstrapped (sampled with replacement) gene sets of the same size from the same gene expression matrix. We also examined the expression levels of genes whose orthologs are annotated as being expressed in zebrafish pharyngeal teeth (www.zfin.org). Within this gene set, 27 of 40 genes were significantly more highly expressed in at least one freshwater population, with no genes expressed significantly higher (as determined by cuffdiff2 [47,53–55], see Materials and Methods) in marine samples than either freshwater population (Fig 2D).

### Increased freshwater expression of stem cell maintenance genes

Tooth development is controlled by several deeply conserved developmental signaling pathways [50,52]. To test whether expression changes in the components of specific developmental signaling pathways have evolved in the two high-toothed freshwater populations, we next analyzed the expression levels of stickleback orthologs of genes implicated in mammalian tooth development and annotated as components of different signaling pathways [36]. When comparing gene expression levels in freshwater dental tissue to marine dental tissue, genes annotated as part of the TGF-ß signaling pathway displayed significantly increased expression in freshwater dental tissue (S3A–S3F Fig).

Since these two freshwater populations have a largely different developmental genetic basis for their evolved tooth gain [30], we next asked whether any pathways were upregulated or downregulated specifically in one freshwater population. When comparing the expression of genes in PAXB_FW_ dental tissue to expression in CERC_FW_ or marine dental tissue, genes not only in the TGF-ß pathway, but also in the WNT signaling pathway, displayed significantly increased expression, consistent with the differing genetic basis of tooth gain in these populations (S3B Fig). In contrast, no significant pathway differences were found comparing CERC_FW_ to PAXB_FW_ or marine (S3C Fig).

We next asked whether any pathways, regardless of previous implication in tooth development, were significantly upregulated in either or both freshwater transcriptomes. Genes upregulated in freshwater dental tissue were enriched for Gene Ontology (GO) terms involved in anatomical structure development, signaling, and regulation of cell proliferation (S4A Fig, S6 Table). Genes upregulated in PAXB_FW_ dental tissue over marine were enriched for GO terms involved in cell proliferation, division and cell cycle regulation, as well as DNA replication (S4B Fig, S7 Table), while genes upregulated in CERC_FW_ over marine were enriched for GO terms involved in cell locomotion, movement, and response to lipids (S4C Fig, S8 Table). 204 of the 454 and 432 GO terms that were enriched in genes upregulated in PAXB_FW_ and CERC_FW_ relative to marine, respectively, were shared, further supporting the convergent gain of freshwater gene expression.

As teeth are constantly being replaced in polyphyodont adult fish, potentially due to the action of dental stem cells [40], we hypothesized that genes involved in stem cell maintenance have evolved increased expression in freshwater tooth plates, given the higher rate of newly forming teeth previously found in adults [30], and the possibly greater number of stem cell niches in high-toothed fish. We further hypothesized that since teeth are developmentally homologous to hair, perhaps an ancient genetic circuit regulating vertebrate placode replacement controls both fish tooth and mammalian hair replacement. For example, the *Bmp6* gene, previously described as expressed in all stickleback teeth [41] was significantly upregulated in CERC_FW_ fish, consistent with the evolved major increases in tooth number in this population (S4 Table). In contrast, no such significant upregulation was observed in the expression of PAXB_FW_ *Bmp6* (S4 Table), consistent with the observed evolved *cis*-regulatory decrease in PAXB_FW_ *Bmp6* expression [41]. Further supporting this hypothesis, the expression of the stickleback orthologs of a previously published set of mouse hair follicle stem cell (HFSC) signature genes [56] were significantly upregulated in freshwater dental tissue (S3A Fig), with 84 and 75 out of 254 genes displaying significant increases in expression in PAXB_FW_ and CERC_FW_, respectively. CERC_FW_ dental tissue displayed a small but significant increase in expression of this set of HFSC orthologs relative to both PAXB_FW_ and marine samples (S3C Fig).

In cichlid fish, pharmacology experiments revealed that reductions in tooth density can be accompanied by concomitant increases or decreases in taste bud density [39]. To begin to test whether derived high-toothed stickleback populations have also evolved significantly altered levels of known taste bud marker gene expression, we examined the expression levels of known taste bud markers *Calbindin2* and *Phospholipase Beta 2* [57], as well as taste receptors such as *Taste 1 Receptor Member 1*, *Taste 1 Receptor Member 3*, and *Polycystin 2 Like 1* [58]. Although four of these five genes had detectable significant expression changes between different populations, no consistent freshwater upregulation or downregulation of taste bud marker genes was seen (S5 Fig).

### *Cis* and t*rans* regulatory changes in gene expression

**Fig 3:**
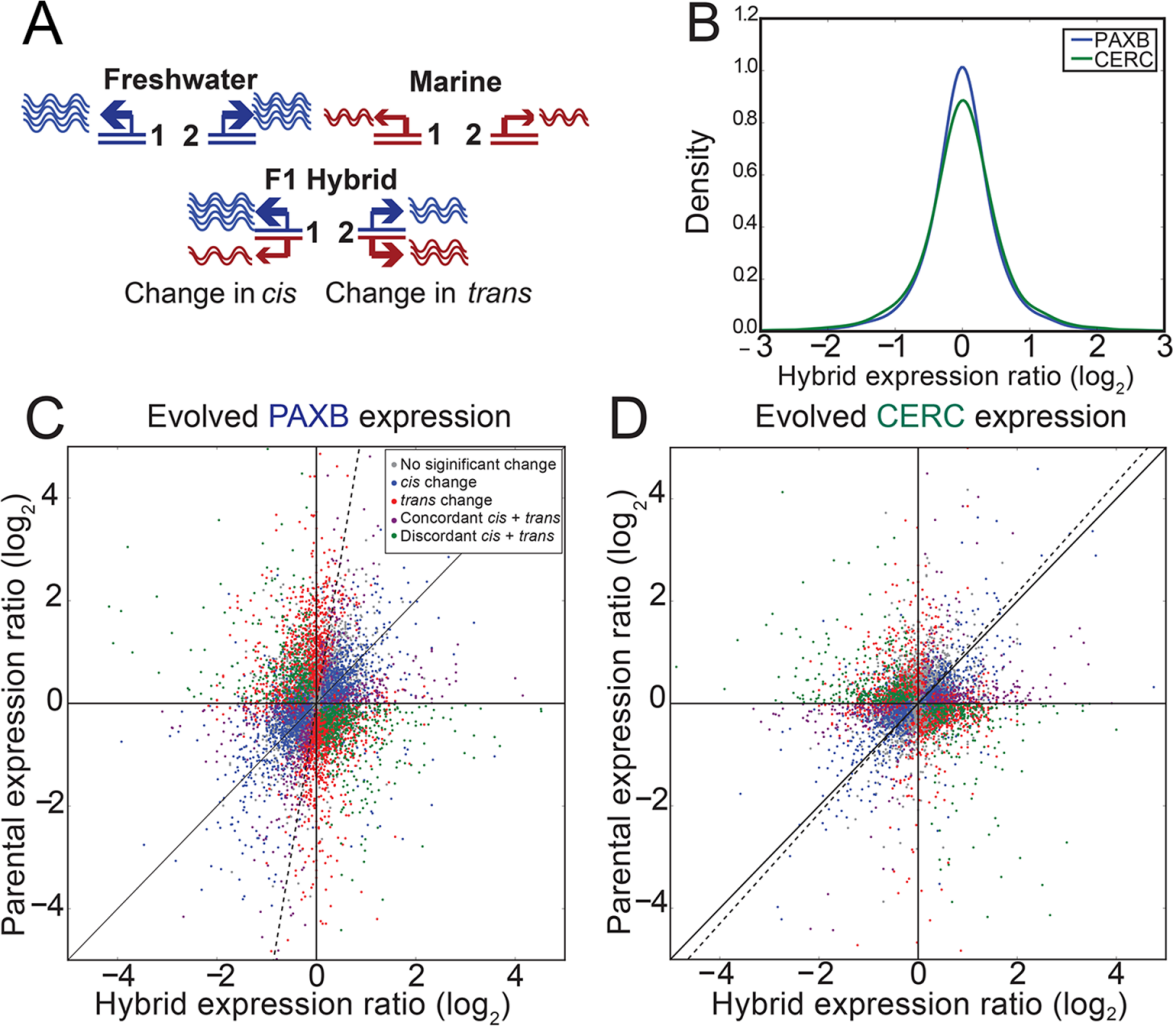
Evolved changes in *cis*-regulation. (A) Cartoon showing the two different regulatory changes detectable by our F1 hybrid system. Both genes 1 and 2 show an evolved increase of expression in freshwater fish, but the freshwater allele of gene 1 but not gene 2 is expressed more highly in F1 hybrids. Therefore, gene 1 has evolved its increased gene expression through *cis*-regulatory changes, while gene 2 was modulated by *trans* regulatory changes. (B) Density plot showing the measured *cis*-regulatory changes. Neither population displayed a significant allelic bias, as measured by a Wilcoxon signed-rank test. (C-D) Gene expression changes in both parental and hybrid dental tissue–genes are color-coded based on the role of *cis* and/or *trans* change in PAXB_FW_ (C) or CERC_FW_ (D) dental tissue. Dashed line indicates the first principal component axis.

Evolved changes in gene expression are due to a combination of *cis* acting changes that are linked to the genes they act on, and *trans* acting changes which usually are genetically unlinked to the gene or genes they regulate. Since the genetic basis of freshwater tooth gain mapped to largely non-overlapping intervals in these two populations [30], we hypothesized that the observed shared freshwater gene expression changes were the result of a similar *trans* environment, but a largely different set of *cis* changes. To test this hypothesis, we measured evolved *cis* expression changes in marine-freshwater F1 hybrids, which have marine and freshwater alleles present in the same *trans* environment. We raised both CERC_FW_-marine and PAXB_FW_-marine F1 hybrids to the late juvenile stage, dissected their ventral pharyngeal tooth plates, then generated and sequenced five barcoded RNA-seq libraries per population (10 total). We then quantified the *cis* expression change as the ratio of the number of reads mapping uniquely to the freshwater allele of a gene to the number of uniquely mapping marine reads (Fig 3A, S9–S11 Tables). *Trans* expression changes were calculated by factoring the *cis* change out from the overall parental expression change [19].

We found 11,832 and 8,990 genes in PAXB_FW_ and CERC_FW_ F1 hybrids, respectively, that had a fixed marine-freshwater sequence difference which had more than 20 total reads mapping to it. We observed no significant bias towards either the marine or freshwater allele in either set of F1 hybrids (Fig 3B). We next classified genes into one of four categories (*cis* change only, *trans* change only, concordant *cis* and *trans* changes, discordant *cis* and *trans* changes). We found 1640 and 1116 PAXB_FW_ (Fig 3C) and CERC_FW_ (Fig 3D) genes, respectively, with only significant *cis* changes, and 1873 and 1048 genes, respectively, with only significant *trans* changes. We also found 478 and 359 genes with significant *cis* and *trans* changes in the same direction, which we term concordant changes in gene expression. Conversely, we found 772 and 607 genes with significant *cis* and *trans* changes in opposing directions, which we termed discordant changes. Discordant *cis* and *trans* changes were more common in both populations, suggesting selection for stable levels of gene expression.

### *Trans* regulatory changes dominate

**Fig 4:**
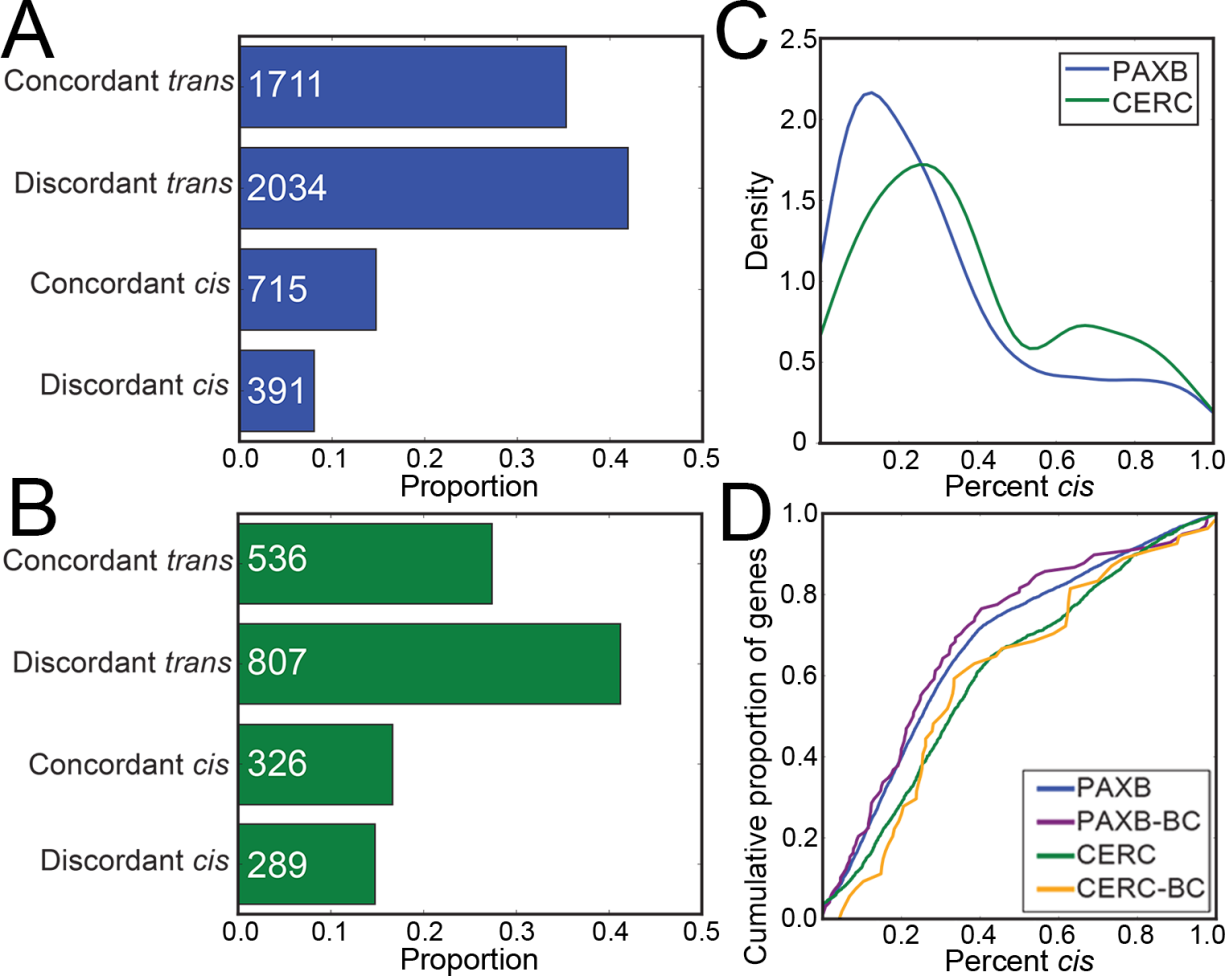
*Trans* changes predominate evolved dental gene expression changes. (A-B) Proportion of differentially expressed genes displaying opposing and concordant *cis* and *trans* changes in PAXB_FW_ (A) or CERC_FW_ (B) dental tissue. Genes whose expression differences were mostly explained by *cis* changes tended to be more concordant (*P* = 5.0e-17, 0.002 for PAXB_FW_ and CERC_FW_, respectively) than those mostly explained by *trans* changes. (C) Density of the relative percentage of gene expression differences which are explained by *cis* changes in PAXB_FW_ and CERC_FW_ dental tissue. (D) Cumulative percentage of percentage of gene expression due to *cis* changes. Genes in CERC_FW_ samples display a higher percentage *cis* change than genes in PAXB_FW_ samples (*P* = 1.25e-22, Mann-Whitney U test).

We next wanted to determine the relative contribution of *cis* and *trans* gene expression changes to evolved changes in gene expression. We restricted our analysis to differentially expressed genes (as determined by cuffdiff2 [47]) to examine only genes with a significant evolved difference in gene expression and quantifiable (i.e. genes with transcripts containing a polymorphic variant covered by at least 20 reads) *cis* and *trans* expression changes. When evolving a change in gene expression, the *cis* and *trans* regulatory basis for this change can be concordant (*cis* and *trans* effects both increase or decrease expression) or discordant (*cis* effects increase and *trans* decrease or vice versa). We hypothesized that genes would tend to display more discordant expression changes, as stabilizing selection has been found to buffer gene expression levels [17,22,59]. To test this hypothesis, we binned differentially expressed genes into a 2x2 contingency table, with genes classified as *cis* or *trans* based on which effect controlled the majority of the evolved expression change, and discordant or concordant based on the direction of the *cis* and *trans* changes (Fig 4A and 4B). In the CERC_FW_ population, significantly more discordant changes than expected by a neutral mode*l* (*P* = 1.35e-7, binomial test) have evolved. In both populations, we found increased discordant changes when the *trans* effect is larger than the *cis* effect (*P* = 1.29e-7, 1.44e-13, PAXB_FW_ and CERC_FW_ respectively, binomial test). In both populations, we observe the opposite (an enrichment of concordant changes) when the *cis* effect is stronger, relative to the ratio when the *trans* effect is dominant (*P* = 1.34e-36, 8.2e-11 PAXB_FW_ and CERC_FW_ respectively, binomial test). When considering all (not just differentially expressed) genes with quantifiable *cis* and *trans* expression changes, discordant changes dominated regardless of the relative strength of the *cis* effect (S6 Fig).

If all gene expression changes were due to changes only in *cis*, we would expect to see the measured *cis* ratios in the hybrids match the parental expression ratios. Instead, in both cases of evolved change, we saw parental expression ratios of a greater magnitude than F1 hybrid ratios, indicating a stronger contribution of *trans* changes to overall gene expression changes (Fig 3C and 3D). Indeed, when we examined the overall percentage of expression changes of differentially expressed genes that were due to changes in *cis*, we observed median per gene values of only 25.2% and 32.5% of PAXB_FW_ and CERC_FW_ gene expression changes, respectively (Fig 4C). Comparing the expression levels of orthologs of known dentally expressed genes from the BiteIt [48] and ToothCODE [36] databases revealed a similarly small number of gene expression changes explained by changes in *cis*, relative to the genome-wide average (Fig 4D). Evolved changes in CERC_FW_ gene expression were more due to changes in *cis* than PAXB_FW_ genes (Fig 4D, *P* = 1.25e-22, Mann-Whitney U test). Thus, *trans* effects on gene expression dominate the evolved freshwater gene expression changes.

### *Trans* regulatory changes are more likely to be shared between freshwater populations

**Fig 5:**
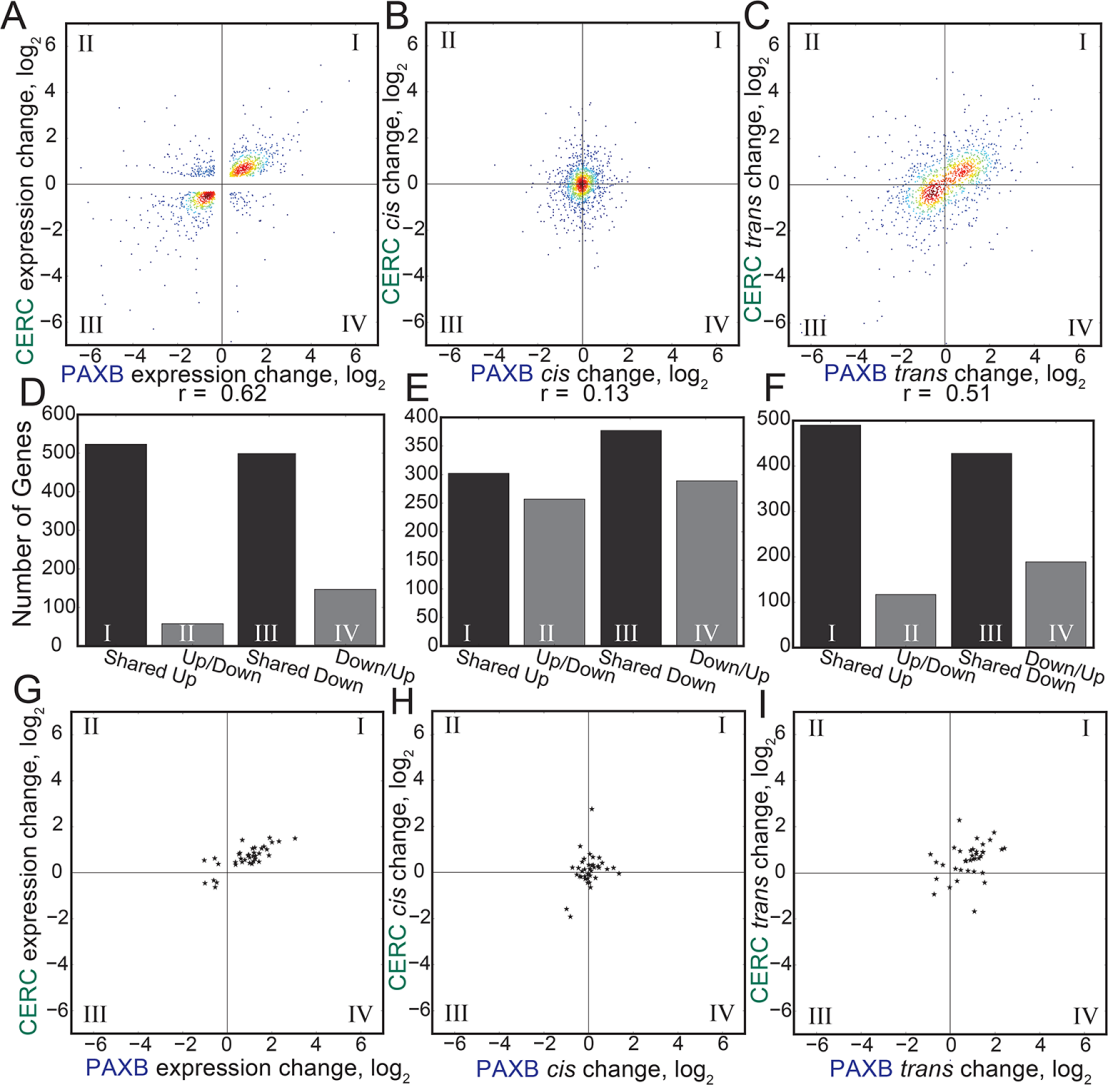
*Trans* changes are more likely to be shared across populations. (A) Genes with significantly different evolved expression in both freshwater populations relative to marine fish, showing significantly correlated changes in gene expression in PAXB_FW_ and CERC_FW_ dental tissue. (B) Freshwater dental tissue had a significant but small number of shared *cis*-regulatory changes. (C) Freshwater dental tissue showed significantly correlated changes in *trans* expression changes. A-C show genes with significant expression changes between populations and quantifiable (i.e. genes with transcripts containing a polymorphic SNP covered by at least 20 reads) *cis*-regulatory changes in both populations. Density (color) was estimated with a Gaussian kernal density estimator. (D-F) Bar graphs show the number of genes with shared or divergent expression patterns from the above panels. (G-I) Similar to (A-C), but showing only genes in the BiteCode gene set.

We next wanted to test the hypothesis that the shared freshwater gene expression changes were primarily due to shared *trans* changes, rather than shared *cis* changes. We first compared the overall expression levels of genes called differentially expressed between PAXB_FW_ and marine as well as CERC_FW_ and marine. We restricted our analysis to differentially expressed genes whose *cis*-regulatory change we were able to measure in our F1 hybrids, including genes without a significant *cis* change. Similar to the genome-wide comparison, we found a highly significant non-parametric correlation coefficient (Spearman's r = 0.62, *P* = 1.2e-132) for the expression change of these shared differentially expressed genes (Fig 5A). When comparing the PAXB_FW_ *cis* changes of these genes to the CERC_FW_ *cis* changes, however, we found a much lower (though still significant) correlation coefficient (Spearman's r = 0.13, *P* = 5.1e-6) (Fig 5B). We calculated *trans* changes for each of these differentially expressed genes, defined as the difference between the expression change in the freshwater parent relative to marine and the freshwater allele relative to the marine in the F1 hybrid [18,19,60]. When comparing the calculated *trans* changes for these shared differentially expressed genes, we observed much higher correlation coefficient (Spearman's r = 0.51, *P* = 1.2e-80) (Fig 5C). When comparing all, not just differentially expressed, genes, *trans* changes are still likely to be more shared than *cis* (S7 Fig). Additionally, 35/38 of the shared differentially expressed putative dental genes have shared regulatory increases or decreases in both freshwater populations relative to marine in overall expression difference. 32/38 of these gene show regulatory changes in the same direction in *trans*, but only 25/38 in *cis* (Fig 5G–5I). Thus, the *trans* effects on evolved gene expression are more likely to be shared by both freshwater populations than the *cis* changes.

## Discussion

We sought to test the relative contribution of *cis* and *trans* gene regulatory changes during convergent evolution of tooth gain, as well as to ask whether the same or different regulatory changes underlie evolved changes in gene expression during this case of convergent evolution. We quantified the overall regulatory divergence, as well as the specific contribution of *cis* and *trans* changes, between ancestral low-toothed marine and two different independently derived populations of high-toothed freshwater sticklebacks. Similar overall changes in gene expression have evolved in both freshwater populations, especially in orthologs of known dental regulators in mammals. In this system, *trans*-regulatory changes play a larger role than *cis* changes in both populations. Furthermore, *trans* acting changes were much more likely to be shared between freshwater populations than *cis* changes, suggesting the two high-toothed populations evolved their similar gene expression patterns through independent genetic changes.

### Convergent evolution of dental gene expression

Convergent evolution at the gene expression level occurs when similar gene expression levels evolve in different populations. Both the PAXB_FW_ and CERC_FW_ stickleback populations have adapted from an ancestral marine form to their current freshwater environments. The genomic nature of their derived changes appears largely divergent, with major axis of variation separating PAXB_FW_ genomes from the geographically proximal marine populations (LITC_M_), as well as the more distant marine (RABS_M_) and CERC_FW_ populations. However, when looking at the gene expression basis of their convergently evolved gain in tooth number, orthologs of genes implicated in mammalian dental development showed strong correlated freshwater gains in expression. This correlation suggests both that sticklebacks deploy conserved genetic circuits regulating tooth formation during tooth replacement, but also that both populations have convergently evolved changes to similar downstream transcriptional circuits resulting in a gain of tooth number.

Though both freshwater populations showed strongly correlated changes in evolved gene expression at the *trans* regulatory level, the *cis* changes were largely not shared across populations. This was especially true for putative dentally expressed genes with evolved expression changes–the vast majority of the *trans* but not *cis* expression changes were shared between both freshwater populations. This suggests that the similar freshwater gene expression patterns evolved through independent genetic changes. It is possible that the small number of shared *cis* changes are sufficient to drive the observed changes to the overall *trans* regulatory environments. However previous work has shown that the genetic basis of tooth gain in these two populations is largely distinct [30], and it seems parsimonious that the genetic basis of a gain in dental gene expression is also mostly independent. Thus, convergent freshwater gene expression changes appear to be largely due to distinct, independent population-specific regulatory changes. This finding suggests that there are many regulatory alleles that are accessible during the evolution of an adaptive trait.

### *Trans* effects dominate

Other studies have used RNA-seq to compare the relative contribution of *cis* and *trans*-regulatory changes in the evolution of gene expression in a multitude of species and tissues. In mice, evolved gene expression changes in the liver [18] and the retina [61] were driven primarily by *cis*-regulatory changes. In *Drosophila*, work on organismal-wide evolved gene expression changes on the genome-wide level has shown the opposite, with *trans*-regulatory effects playing a larger role in the evolution of gene expression [19,22]. Other studies have found *trans* effects contribute more to intraspecific comparisons, while *cis* effects contribute more to interspecific comparisons [17,20,60]. Consistent with this, we observe *trans* effects dominating in both of our intraspecific comparisons.

Another key distinction could be that *cis*-regulatory effects dominate when looking at more cellularly homogenous tissues, while *trans*-regulatory effects dominate when looking at more heterogeneous tissues. Stickleback tooth plates likely fall into an intermediate category, less heterogenous in cell type composition than a full adult fly or fly head, but more heterogeneous than a specialized tissue such as the mouse retina. Overall, freshwater tooth plates are more morphologically similar to each other than marine, with freshwater tooth plates possessing a larger area, increased tooth number, and decreased intertooth spacing [30,41]. Freshwater tooth plates likely have more similar cell type abundances and compositions (e.g. more developing tooth germs with inner and outer dental epithelia, and odontogenic mesenchyme) compared to each other than to marine tooth plates. Similar cell types tend to have similar gene expression patterns, even when compared across different species [62]. Much of the shared freshwater increase in dental gene expression could be due to an increase in dental cell types in both freshwater populations. As other evolved changes to stickleback morphology have been shown to be due to *cis* regulatory changes to key developmental regulatory genes [8,33,41,63], this *trans* regulatory increase in cell type abundance could be due to a small number of *cis* regulatory changes. These initially evolved developmental regulatory changes could result in similar downstream changes in the developmental landscape, resulting in the shared increase in dental cell types. Consistent with this interpretation, stickleback orthologs of genes known to be expressed during mammalian tooth development were found here to have a much greater incidence of convergently evolved increase in *trans* regulatory gene expression.

### Compensatory *cis* and *trans*

Previous studies [17,18] have shown compensatory *cis* and *trans* changes are essential for the evolution of gene expression. These findings are consistent with the idea that the main driving force in the evolution of gene expression is stabilizing selection [59] where compensatory changes to regulatory elements are selected for to maintain optimal gene expression levels. In both PAXB_FW_ and CERC_FW_ dental tissue, when considering all genes with a quantifiable (i.e. polymorphic and covered by ~20 reads, see Methods) *cis* effects, discordant compensatory *cis* and *trans* changes were far more common than concordant ones. This trend could be driven by some initial selection on pleiotropic *trans* changes, followed by selection for compensatory *cis* changes to restore optimal gene expression levels [17,18,22]. However, the *trans*, but not the *cis*, evolved changes in gene expression were highly shared among the two freshwater populations. Thus, collectively our data support a model where two independently derived populations have convergently evolved both similar genome-wide expression levels as well as ecologically relevant morphological changes through different genetic means.

### Potential parallels between teeth and hair regeneration

PAXB_FW_ and CERC_FW_ sticklebacks have an increased rate of new tooth formation in adults relative to their marine ancestors [30]. In constantly replacing polyphyodonts, it has been proposed that teeth are replaced through a dental stem cell intermediate [37,38]. A strong candidate gene underlying a large effect PAXB_FW_ tooth quantitative trait locus (QTL) is the secreted ligand *Bone Morphogenetic Protein 6* (*Bmp6*) [41], which is also a key regulator of stem cells in the mouse hair follicle [56]. Freshwater dental tissue displayed significantly increased expression of known signature genes of mouse hair follicle stem cells, perhaps reflecting more stem cell niches supporting the higher tooth numbers in freshwater fish. Genes upregulated in freshwater dental tissue also were significantly enriched for GO terms involved in the cell cycle and cell proliferation. Together these findings suggest that both freshwater populations have evolved an increased tooth replacement rate through an increased activity or abundance of their dental stem cells, and also suggest the genetic circuitry regulating mammalian hair and fish tooth replacement might share an ancient, underlying core gene regulatory network.

## Materials and methods

### Ethics statement

Experiments were approved by the Institutional Animal Care and Use Committee of the University of California-Berkeley (protocol # R330).

### Stickleback husbandry

Fish from all populations were raised in 110L aquaria in brackish water (3.5g/L Instant Ocean salt, 0.217mL/L 10% sodium bicarbonate) at 18°C in 8 hours of light per day. Young fry [standard length (SL) < 10 millimeters (mm)] were fed a diet of live *Artemia*, early juveniles (SL ~10–20 mm) a combination of live *Artemia* and frozen *Daphnia*, and older juveniles (SL > ~20 mm) and adults a combination of frozen bloodworms and *Mysis* shrimp.

### Skeletal staining and imaging

Sticklebacks were fixed in 10% neutral buffered formalin overnight at 4°C. Fish were washed once with water and then stained in 1% KOH, 0.008% Alizarin Red for 24 hours. Following a water rinse, fish were cleared in 0.25% KOH, 50% glycerol for 2–3 weeks. Branchial skeletons were dissected as previously described [64]. Pharyngeal teeth were quantified with fluorescent illumination using a TX2 filter on a Leica DM2500 microscope. Representative tooth plates were created using montage z-stacks on a Leica M165 FC using the RhodB filter. Adult fish were imaged using a Canon Powershot S95. Some tooth count data from the CERC_FW_, RABS_M_, and PAXB_FW_ populations; n = 11, 13, 29, respectively, (see S1 Table) have been previously published [30].

### DNA preparation and genome resequencing

Caudal fin tissue was placed into 600μl tail digestion buffer [10mM Tris pH 8.0, 100mM NaCl, 10mM EDTA, 0.05% SDS, 2.5μl ProK (Ambion AM2546)] for 12 hours at 55°C. Following addition of 600 μl of 1:1 phenol:chloroform solution and an aqueous extraction, DNA was precipitated with the addition of 1ml 100% ethanol, centrifuged, washed with 75% ethanol, and resuspended in water. 50ng of purified genomic DNA was used as input for the Nextera Library prep kit (Illumina FC-121-1031), and barcoded libraries were constructed following the manufacturer’s instructions. Library quality was verified using an Agilent Bioanalyzer. Libraries were pooled and sequenced on an Illumina HiSeq 2000 (see S2 Table for details), resulting in a mean of 52.8 million reads per sample, with a max of 70.3 million reads and a minimum of 39 million reads (S2 Table).

### RNA purification and creation of RNA-seq libraries

Late juvenile stage female sticklebacks (SL ~40mm) were euthanized in 0.04% Tricaine. Dissected [64] bilateral ventral pharyngeal tooth plates were placed into 500μl TRI reagent, then incubated at room temperature for 5 minutes. Following addition of 100μl of chloroform, a further 10 minute incubation and centrifugation, the aqueous layer was extracted. Following addition of 250μl isopropyl alcohol and 10 minute incubation, RNA was precipitated by centrifugation, washed with 75% EtOH, and dissolved in 30ul of DEPC-treated water. RNA integrity was assayed by an Agilent Bioanalyzer. 500ng of RNA from each fish was used as input to the Illumina stranded TruSeq polyA RNA kit (Illumina RS-122-2001), and libraries were constructed following the manufacturer’s instructions. Library quality was analyzed on an Agilent Bioanalyzer, and libraries were pooled and sequenced on an Illumina HiSeq2000 (see S3 Table). We obtained a mean of 84.1 million reads among the parental samples, with a max of 91.0 million and a minimum of 78.6 million (S3 Table).

### Gene expression quantification and analysis

RNA-seq reads were mapped to the stickleback reference genome [31] using the STAR aligner [65] (version 2.3, parameters = —alignIntronMax 100000—alignMatesGapMax 200000—outFilterMultimapNmax 20—outFilterMismatchNmax 999—outFilterMismatchNoverLmax 0.04—outFilterType BySJout), using ENSEMBL genes release 85 as a reference transcriptome. The resulting SAM files were sorted and indexed using Samtools version 0.1.18 [66], PCR duplicates were removed, read groups added and mate pair information fixed using Picard tools (version 1.51) (http://broadinstitute.github.io/picard/) with default settings. Gene expression was quantified with the Cufflinks suite (v 2.2.1) [47,53–55] using ENSEMBL genes as a reference transcriptome, with gene expression quantified with cuffquant (-u—library-type fr-firststrand) and normalized with cuffnorm. Differentially expressed genes were found using cuffdiff2, with parameters (-u—FDR .1—library-type fr-firststrand, using the reference genome for bias correction). Genes with a mean expression less than 0.1 FPKM were filtered from further analysis.

### Gene set and gene ontology enrichment

The BiteCode gene set was generated by combining all genes in the BiteIt (http://bite-it.helsinki.fi/) or ToothCODE (http://compbio.med.harvard.edu/ToothCODE/) [36] databases. Stickleback orthologs or co-orthologs were found using the annotated names of ENSEMBL stickleback genes. Gene set expression change statistical enrichment was done as previously described [67]. Briefly, a t-test was performed for each gene to test for a difference in mean expression between the two treatments. The resulting t-values were subject to a 1-sample t-test, with the null model that the mean of the t-values was 0. Cutoffs were validated using 10,000 bootstrapped replicate gene sets drawn from the same gene expression matrix. Stickleback orthologs of mouse or human genes were determined using annotated ENSEMBL orthologs. Sorted lists of genes, ranked by log_2_ expression change in PAXB_FW_ dental tissue relative to marine, CERC_FW_ relative to marine, or the mean of CERC_FW_ and PAXB_FW_ relative to marine, were generated using the measured gene expression data. Gene Ontology enrichment was done using Gorilla [68,69], and results were visualized using REVIGO [70].

### Detection of genomic and transcriptomic variants

Genomic resequencing reads were aligned to the stickleback reference genome [31] using the bwa aln and bwa sampe modules of the Burrows-Wheeler Alignment tool (v 0.6.0-r85) [71]. Resulting SAM files were converted to BAM files, sorted and indexed by Samtools version 0.1.18 [66], with PCR duplicates removed by Picard tools. GATK's (v3.2–2) IndelRealigner (parameter: '-LOD 0.4'), BaseRecalibrator, and PrintReads were used on the resulting BAM files. BAM files from the above RNA-seq alignment were readied for genotype calling using GATK's SplitNCigarReads, BaseRecalibrator, and PrintReads. Finally, the UnifiedGenotyper was used to call variants from the RNA-seq and DNA-seq BAM files, with parameters (-stand_call_conf 30 -stand_emit_conf 30 -U ALLOW_N_CIGAR_READS—genotype_likelihoods_model BOTH) [43,45]. This analysis identified a set of 8,341,326 variants.

Principal components analysis of the genome-wide set of variants was performed by first filtering all multiallelic variants or variants with a missing genotype, resulting in a set of 1,690,729 variants. PCA was performed using FactoMiner [46] and a set of custom R scripts. Phylogenetic trees were constructed using the set of variants, downsampled to 67,507 SNPs (no indels) for use with BEAST and SNAPP [72,73]. We constructed phylogenies using SNAPP, estimating substitution rate and proportion invariant from the data, and ran 1 million generations of MCMC simulations. The best tree was picked with TreeAnnotator and visualized with FigTree.

To accurately phase RNA-seq data from F1 hybrids, pseudo-transcriptomes were created for each hybrid. The pseudo-transcriptomes consist of the predicted sequence for each allele within an F1 hybrid, with all predicted splicing variants of a gene collapsed to a single transcript. A variant was added to the pseudo-transcriptome if and only if it was homozygous in the sequenced parents (or parent’s sibling in the case of the RABS_M_ parent of the CERC_FW_ x RABS_M_ F1 hybrids) and called heterozygous in the F1 hybrid.

### *Cis* and *trans* regulatory divergence quantification

RNA-seq reads from F1 hybrid sticklebacks were aligned to the individual’s pseudo-transcriptome using STAR (v 2.3) with the parameters:—outFilterMultimapNmax 1 and—outFilterMultimapScoreRange 1. By only looking at uniquely aligning reads, we ensured we only considered reads which overlapped a heterozygous variant site. Counting these unique reads minimizes double counting a single read that supports two different variant positions. Total *cis* divergence in each F1 hybrid was quantified by comparing the number of reads mapping uniquely to each allele in the pseudo-transcriptome.

Following *cis* divergence quantification in all F1 hybrids, we considered the overall *cis* change in the different freshwater populations. Genes which only had 20 or fewer uniquely mapping reads across all replicates were filtered from further analysis. We filtered 28 genes that had >32 fold expression changes that included genes that either had zero reads from one allele and thus infinite expression differences (20 genes), were highly repetitive (2 genes), or mitochondrial (2 genes). Reported *cis* ratios were calculated by comparing the ratio of uniquely mapped freshwater reads to uniquely mapped marine reads. Evolved *trans* changes were quantified as the difference between the log of the overall gene expression change between the freshwater and marine parents and the log of measured *cis* freshwater expression change. Percent *cis* change was calculated as the absolute value of the log of the *cis* change divided by the sum of the absolute value of the log of the *cis* change and the absolute value of the log of the *trans* change. Statistical significance of *cis* changes was determined by a binomial test comparing overall reads mapping to the freshwater allele to a null model of no *cis* divergence, with a false discovery rate of 1% applied using the Benjamini-Hochberg method. Statistical significance of *trans* changes was determined by a G-test, comparing the expected (based on the measured *cis* change) and observed ratios of marine and freshwater, with a 1% false discovery rate.

## Supporting information

S1 Fig Independent freshwater evolutionary history. (A) Genome-wide phylogeny created from genomic resequencing data. Wild-caught fish are non-italicized. All nodes have 100% posterior probability. Scale bar shows 3% sequence divergence at variant positions. (B) Principal component analysis of genome-wide genotypes separates marine and CERC_FW_ populations from the PAXB_FW_ lake population, with the 2^nd^ PC separating marine and freshwater populations.
(TIF)

S2 Fig Freshwater upregulation of putative dental genes. (A) PAXB_FW_ upregulation of BiteCode genes (282 expressed orthologs, *P* = 9.8e-3, GSEA). (B) CERC_FW_ upregulation of BiteCode genes (*P* = 2.1e-5, GSEA). (C) PAXB_FW_ and CERC_FW_ upregulation of BiteCode genes (*P* = 5.1e-6, GSEA).
(TIF)

S3 Fig Concerted changes in stem cell markers and signaling pathways. (A-F) Changes in gene expression changes of genes annotated as components of the indicated signaling pathways (BMP, FGF, SHH, WNT, ACT, TGFB, NOTCH, or EDA, containing 59, 60, 28, 75, 19, 11, 12, and 6 expressed orthologs, respectively) [36] or orthologs of a described set of mouse hair follicle stem cell signature genes (HFSC, containing 254 expressed orthologs) [56]. Violin plots show the mean expression change of genes in the pathway. (A) Change in freshwater (PAXB_FW_ + CERC_FW_) relative to marine. (B) PAXB_FW_ specific changes (PAXB_FW_ relative to CERC_FW_ + marine). In the WNT and TGFB pathway, 22/75 and 6/11 genes had significantly increased expression respectively (C) CERC_FW_ specific changes (CERC_FW_ relative to PAXB_FW_ + marine). (D) PAXB_FW_ evolved changes (PAXB_FW_ relative to marine) (E) CERC_FW_ evolved changes (CERC_FW_ relative to marine) (F) PAXB_FW_ vs CERC_FW_ changes (PAXB_FW_ relative to CERC_FW_).
(TIF)

S4 Fig Gene ontology of freshwater upregulated genes. (A-C) GO enrichment of genes upregulated in freshwater (A), PAXB_FW_ (B), or CERC_FW_ (C). GO analysis was performed using Gorilla [68], with the results visualized with Revigo [70].
(TIF)

S5 Fig Expression of taste bud marker genes. Expression levels of known taste bud marker genes in marine, PAXB_FW_ and CERC_FW_ tooth plates as assayed by RNA-seq. * indicates differentially expressed genes. Error bars are standard error of the mean.
(TIF)

S6 Fig Compensatory changes dominate genes with no significant evolved gene expression difference. (A-B) Proportion of genes with quantifiable (i.e. genes with transcripts containing a polymorphic SNP covered by at least 20 reads) hybrid expression displaying opposing and concordant *cis* and *trans* changes in PAXB_FW_ (A) or CERC_FW_ (B) dental tissue. Similar to Fig 5, but here showing all genes, not just genes with significantly different expression levels compared to marine. *Trans* regulatory changes predominate, as do opposing over concordant changes. (C) Density plot of the percentage of gene expression changes explained by *cis*-regulatory changes.
(TIF)

S7 Fig *Trans* changes are more likely to be shared across populations. (A) Expression changes of genes with quantifiable (i.e. genes with transcripts containing a polymorphic SNP covered by at least 20 reads) hybrid expression in both freshwater populations relative to marine fish, showing significantly correlated changes in gene expression in PAXB_FW_ and CERC_FW_ tooth plates. (B) *cis* regulatory changes of genes with quantifiable hybrid expression in freshwater dental tissue overall do not display correlated evolved changes. (C) *trans* regulatory changes of genes with quantifiable hybrid expression in freshwater dental tissue. Density (color) was estimated with a Gaussian kernel density estimator. (D-F) Similar to A-C, but showing only genes in the BiteCode gene set, revealing that these orthologs have evolved highly convergent changes in the two freshwater populations (D), despite non-convergent *cis* regulatory changes (E).
(TIF)

S1 Table Population ventral pharyngeal tooth counts. For each fish, the population, ecotype (freshwater or marine), total ventral pharyngeal tooth number (TVTP), total length (TL), standard length (SL), and whether data has been published [30] is shown.
(XLSX)

S2 Table Genomic DNA sequencing reads. For each fish, population and biological replicate number (Fish), the total number of barcoded reads from each fish (reads), and number of reads that mapped and passed all filters (final mapped) is listed.
(XLSX)

S3 Table RNA-seq reads. For each fish, population of parents and biological replicate number (sample), standard length (SL), total reads (generated by HiSeq2000 over two different runs (run1 and run2)), mapped reads (reads that mapped to the genome), and final reads (excludes reads filtered due to low quality or PCR duplication) is listed.
(XLSX)

S4 Table Overall gene expression in tooth plate. Estimated abundance in in fragments per kilobases per million reads (FPKM) of ENSEMBL genes (rows) in ventral pharyngeal dental tissue from three individual fish from three populations (in columns). Mean expression (in FPKM) is shown after the 3 replicates. Log_2_(Pop1/Pop2) shows the fold-change in log_2_ of the estimated mean expression between the two populations. IsSig(Pop1/Pop2) indicates whether the difference was significant as reported by cuffdiff2.
(XLSX)

S5 Table BiteCode genes in sticklebacks. A list of stickleback orthologs in the BiteIt [48] (http://bite-it.helsinki.fi/) or ToothCODE (http://compbio.med.harvard.edu/ToothCODE/) [36] databases.
(XLSX)

S6 Table GO process upregulated in freshwater. Gene Ontology (GO) term category and name are given in GO term and description, with the p-value, q-value, and relative enrichment within genes upregulated in freshwater dental tissue reported by GOrilla [68].
(XLSX)

S7 Table GO process upregulated in PAXB_FW_. Gene Ontology (GO) term category and name are given in GO term and description, with the p-value, q-value, and relative enrichment within genes upregulated in PAXB_FW_ dental tissue reported by GOrilla [68].
(XLSX)

S8 Table GO process upregulated in CERC_FW_. Gene Ontology (GO) term category and name are given in GO term and description, with the p-value, q-value, and relative enrichment within genes upregulated in CERC_FW_ dental tissue reported by GOrilla [68].
(XLSX)

S9 Table F1 hybrid RNA-seq reads. For each ventral pharyngeal tooth plate (VTP), population of parents and biological replicate number (sample), standard length (SL), total reads (generated by HiSeq2000), mapped reads (reads that mapped to the genome), final reads (excludes reads filtered due to low quality or PCR duplication), and unique reads (reads that mapped uniquely to one haplotype) is listed.
(XLSX)

S10 Table PAXB_FW_ vs marine *cis* divergence. Estimated gene expression change in *cis* in log_2_, PAXB_FW_ vs marine. Name is the reported ENSEMBL gene name. Log_2_(F/M) is the log_2_ of the ratio of freshwater vs marine reads mapping uniquely to the gene.
(XLSX)

S11 Table CERC_FW_ vs marine *cis* divergence. Estimated gene expression change in *cis* in log_2_, CERC_FW_ vs marine. Name is the reported ENSEMBL gene name. Log_2_(F/M) is the log_2_ of the ratio of freshwater vs marine reads mapping uniquely to the gene.
(XLSX)
