# Supplementary figures and images for: Convergent evolution of gene expression in two high-toothed stickleback populations
Source: PLoS Genet. 2018 Jun 13;14(6):e1007443. doi: 10.1371/journal.pgen.1007443 (PMC6016950; doi:10.1371/journal.pgen.1007443)

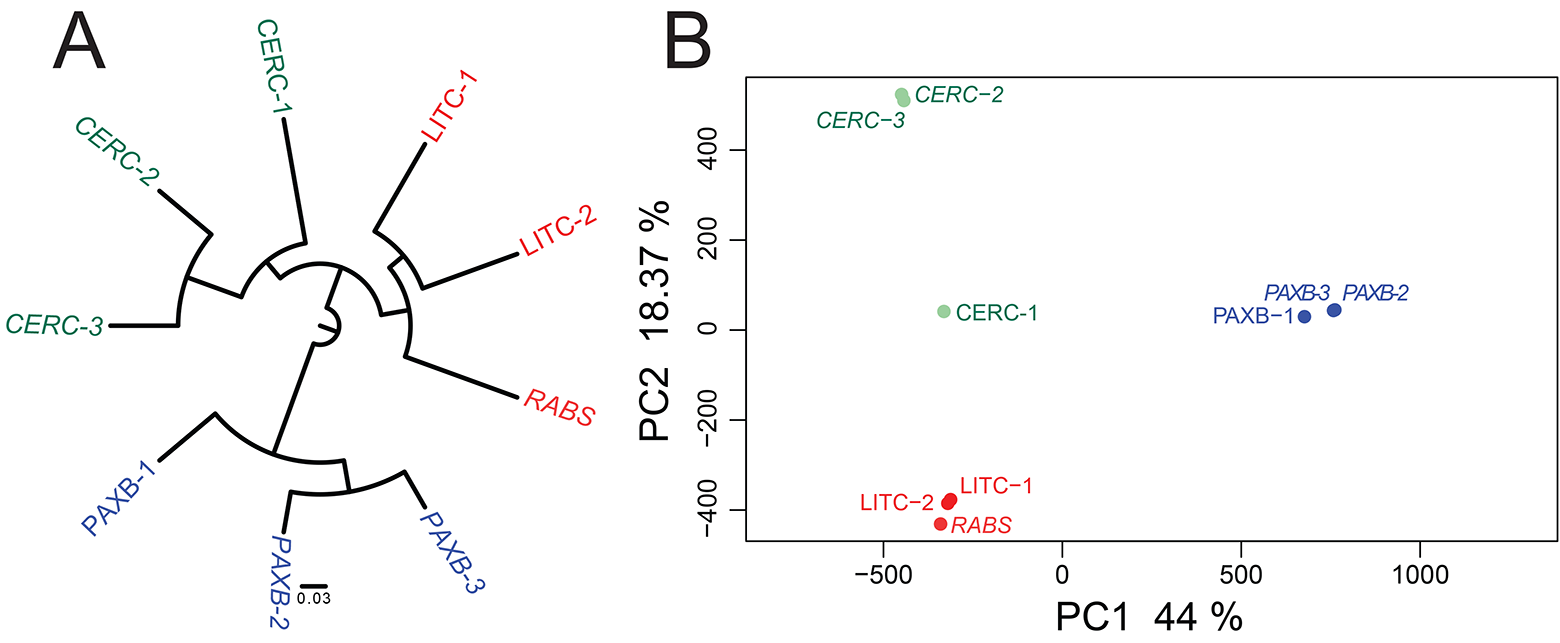

Supplement: S1 Fig — (A) Genome-wide phylogeny created from genomic resequencing data. Wild-caught fish are non-italicized. All nodes have 100% posterior probability. Scale bar shows 3% sequence divergence at variant positions. (B) Principal component analysis of genome-wide genotypes separates marine and CERCFW populations from the PAXBFW lake population, with the 2nd PC separating marine and freshwater populations. (TIF) [file pgen.1007443.s001.tif]

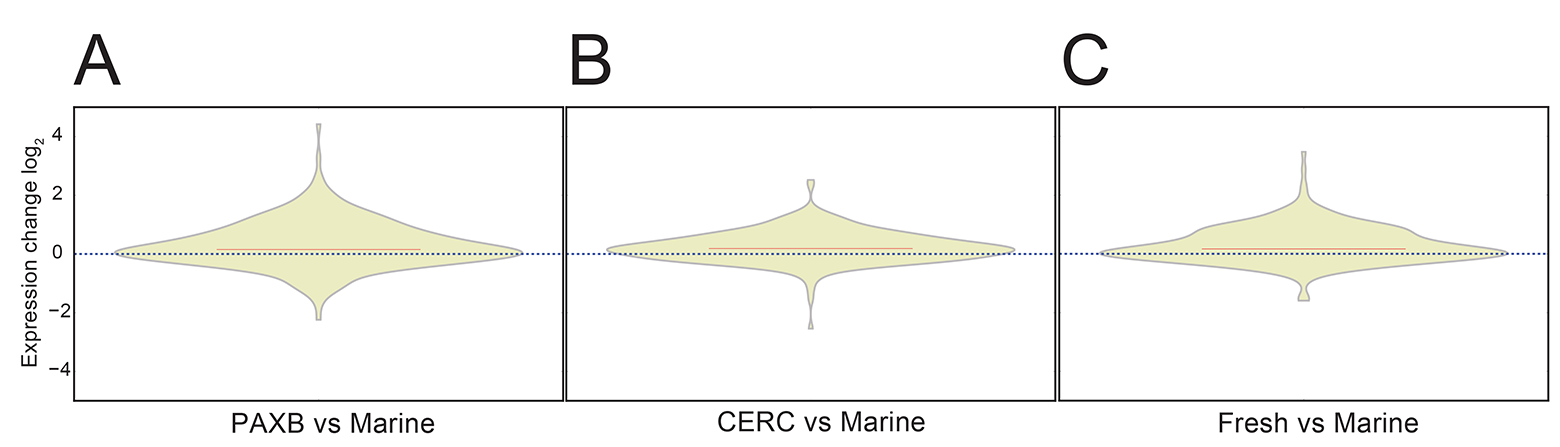

Supplement: S2 Fig — (A) PAXBFW upregulation of BiteCode genes (282 expressed orthologs, P = 9.8e-3, GSEA). (B) CERCFW upregulation of BiteCode genes (P = 2.1e-5, GSEA). (C) PAXBFW and CERCFW upregulation of BiteCode genes (P = 5.1e-6, GSEA). (TIF) [file pgen.1007443.s002.tif]

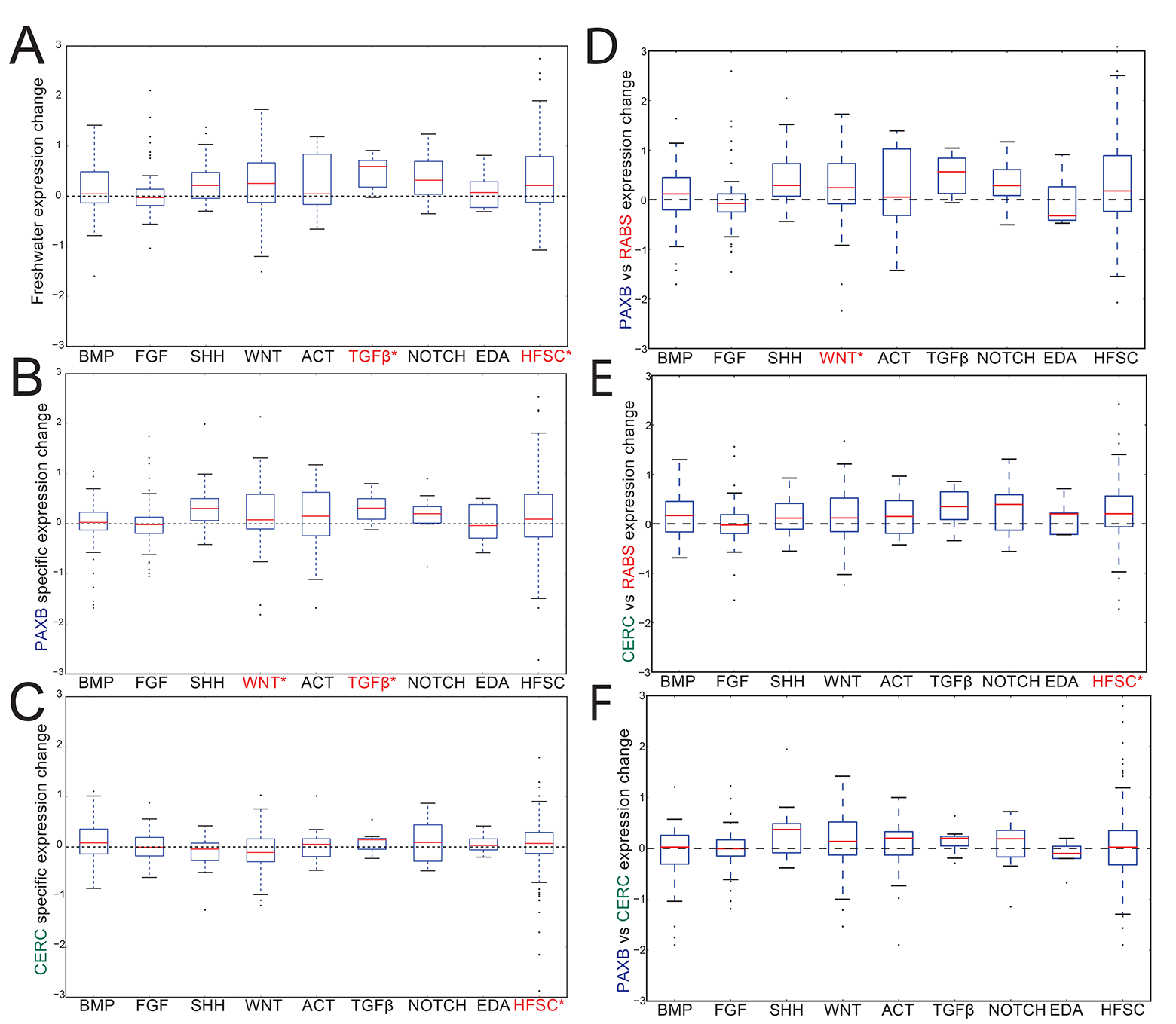

Supplement: S3 Fig — (A-F) Changes in gene expression changes of genes annotated as components of the indicated signaling pathways (BMP, FGF, SHH, WNT, ACT, TGFB, NOTCH, or EDA, containing 59, 60, 28, 75, 19, 11, 12, and 6 expressed orthologs, respectively) [36] or orthologs of a described set of mouse hair follicle stem cell signature genes (HFSC, containing 254 expressed orthologs) [56]. Violin plots show the mean expression change of genes in the pathway. (A) Change in freshwater (PAXBFW + CERCFW) relative to marine. (B) PAXBFW specific changes (PAXBFW relative to CERCFW + marine). In the WNT and TGFB pathway, 22/75 and 6/11 genes had significantly increased expression respectively (C) CERCFW specific changes (CERCFW relative to PAXBFW + marine). (D) PAXBFW evolved changes (PAXBFW relative to marine) (E) CERCFW evolved changes (CERCFW relative to marine) (F) PAXBFW vs CERCFW changes (PAXBFW relative to CERCFW). (TIF) [file pgen.1007443.s003.tif]

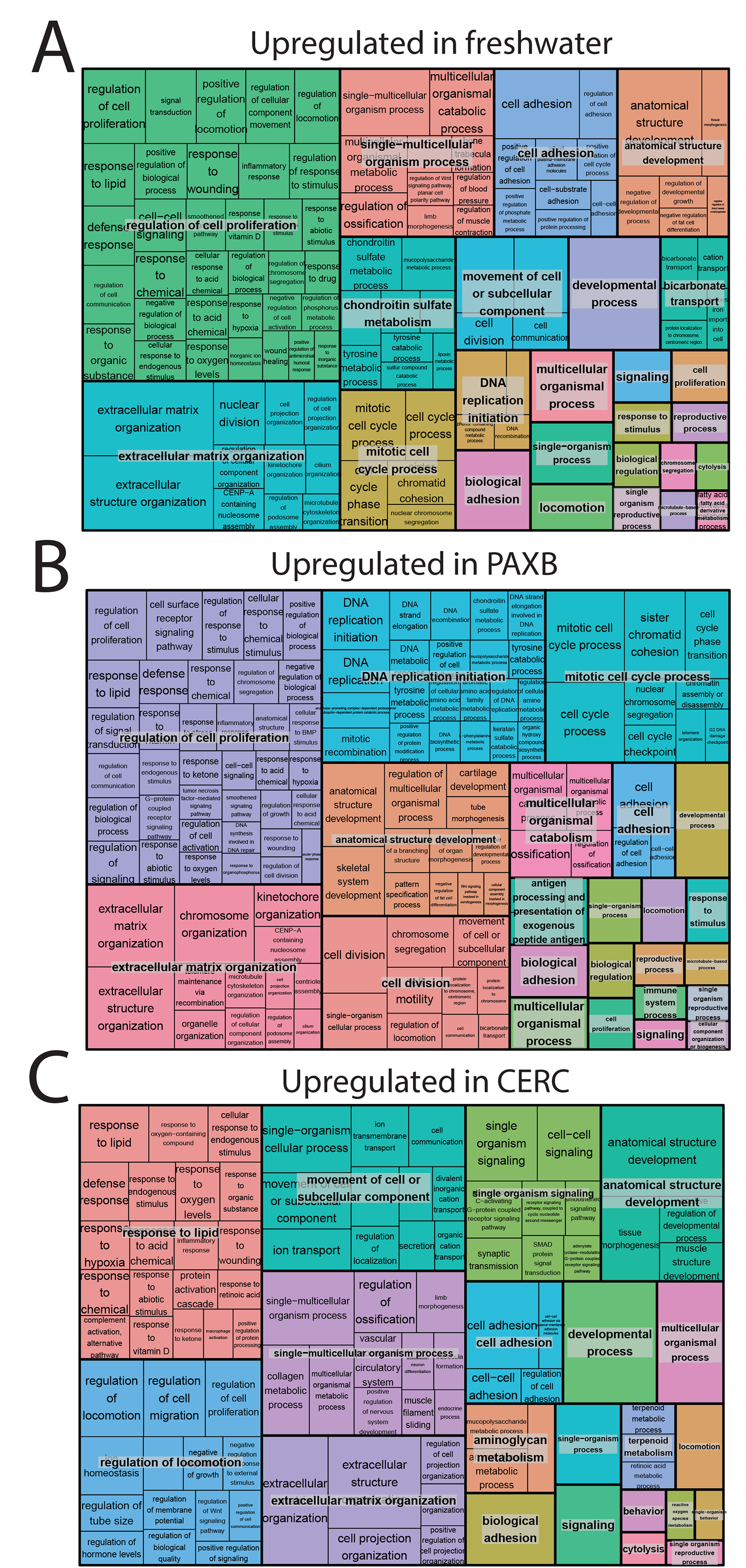

Supplement: S4 Fig — (A-C) GO enrichment of genes upregulated in freshwater (A), PAXBFW (B), or CERCFW (C). GO analysis was performed using Gorilla [68], with the results visualized with Revigo [70]. (TIF) [file pgen.1007443.s004.tif]

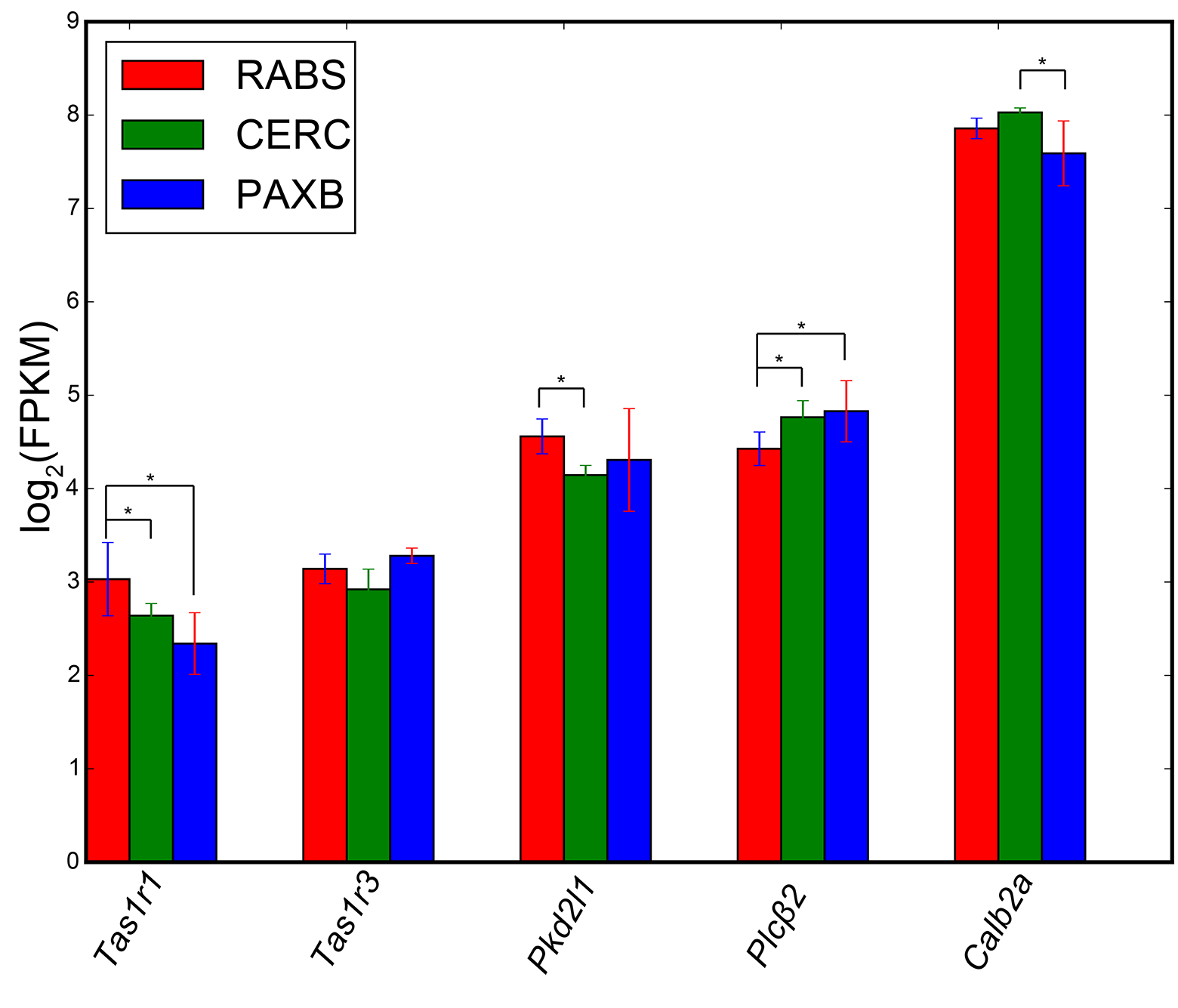

Supplement: S5 Fig — Expression levels of known taste bud marker genes in marine, PAXBFW and CERCFW tooth plates as assayed by RNA-seq. * indicates differentially expressed genes. Error bars are standard error of the mean. (TIF) [file pgen.1007443.s005.tif]

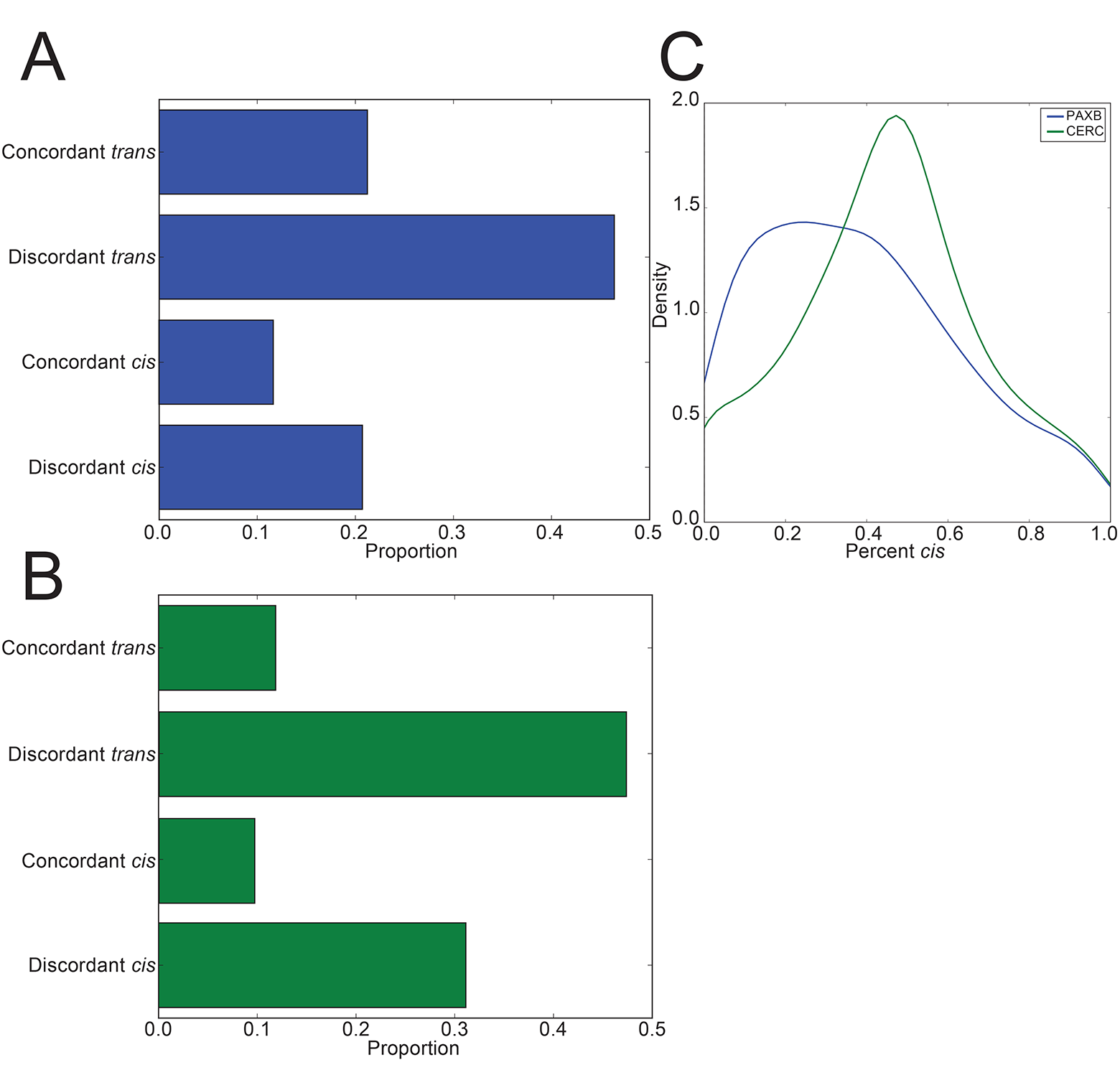

Supplement: S6 Fig — (A-B) Proportion of genes with quantifiable (i.e. genes with transcripts containing a polymorphic SNP covered by at least 20 reads) hybrid expression displaying opposing and concordant cis and trans changes in PAXBFW (A) or CERCFW (B) dental tissue. Similar to Fig 5, but here showing all genes, not just genes with significantly different expression levels compared to marine. Trans regulatory changes predominate, as do opposing over concordant changes. (C) Density plot of the percentage of gene expression changes explained by cis-regulatory changes. (TIF) [file pgen.1007443.s006.tif]

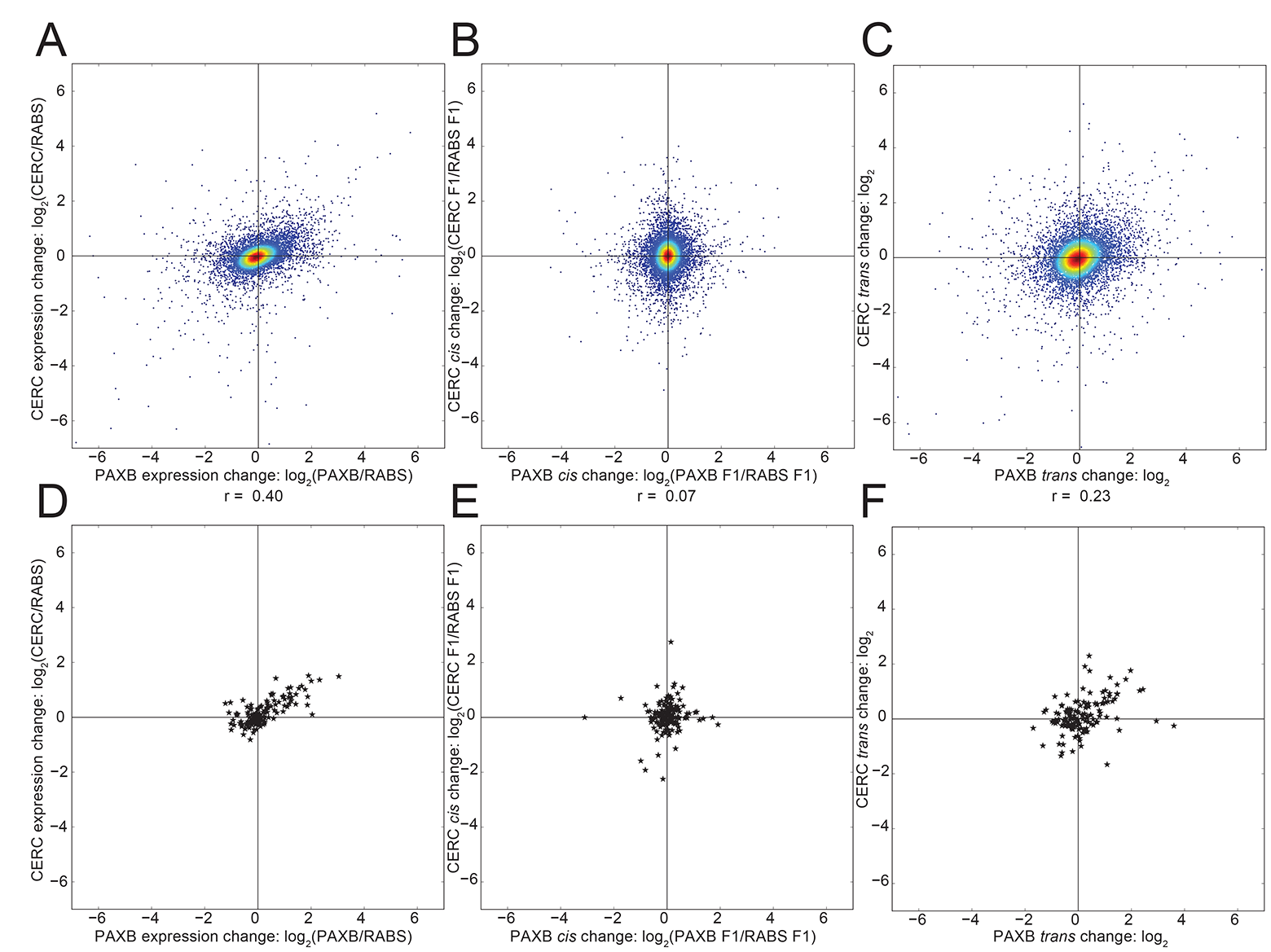

Supplement: S7 Fig — (A) Expression changes of genes with quantifiable (i.e. genes with transcripts containing a polymorphic SNP covered by at least 20 reads) hybrid expression in both freshwater populations relative to marine fish, showing significantly correlated changes in gene expression in PAXBFW and CERCFW tooth plates. (B) cis regulatory changes of genes with quantifiable hybrid expression in freshwater dental tissue overall do not display correlated evolved changes. (C) trans regulatory changes of genes with quantifiable hybrid expression in freshwater dental tissue. Density (color) was estimated with a Gaussian kernel density estimator. (D-F) Similar to A-C, but showing only genes in the BiteCode gene set, revealing that these orthologs have evolved highly convergent changes in the two freshwater populations (D), despite non-convergent cis regulatory changes (E). (TIF) [file pgen.1007443.s007.tif]
